# LPS-Induced Acute Lung Injury: Analysis of the Development and Suppression by the TNF-α-Targeting Aptamer

**DOI:** 10.32607/actanaturae.27393

**Published:** 2024

**Authors:** A. V. Sen’kova, I. A. Savin, E. L. Chernolovskaya, A. S. Davydova, M. I. Meschaninova, A. Bishani, M. A. Vorobyeva, M. A. Zenkova

**Affiliations:** Institute of Chemical Biology and Fundamental Medicine, Siberian Branch of the Russian Academy of Sciences, Novosibirsk, 630090 Russian Federation

**Keywords:** acute lung injury, proinflammatory cytokines, aptamers, target genes

## Abstract

Acute lung injury (ALI) is a specific form of lung inflammation characterized by diffuse alveolar damage, noncardiogenic pulmonary edema, as well as a pulmonary and systemic inflammation. The pathogenesis of ALI involves a cascade inflammatory response accompanied by an increase in the local and systemic levels of proinflammatory cytokines and chemokines. The development of molecular tools targeting key components of cytokine signaling appears to be a promising approach in ALI treatment. The development of lipopolysaccharide (LPS)-induced ALI, as well as the feasibility of suppressing it by an aptamer targeting the proinflammatory cytokine TNF-α, was studied in a mouse model. The TNF-α level was shown to increase significantly and remain steadily high during the development of ALI. LPS-induced morphological signs of inflammation in the respiratory system become most pronounced 24 h after induction. Intranasal administration of TNF-α-targeting aptamers conjugated with polyethylene glycol (PEG-aptTNF-α) to mice with ALI reduced the intensity of inflammatory changes in lung tissue. Assessment of the levels of potential TNF-α target genes (*Usp18*, *Traf1*, and *Tnfaip3*) showed that their expression levels in the lungs increase during ALI development, while declining after the application of PEG-aptTNF-α. Therefore, topical use of TNF-α- targeting aptamers may be an efficient tool for treating ALI and other inflammatory lung diseases.

## INTRODUCTION

Acute lung injury (ALI) and its sequela, acute respiratory distress syndrome (ARDS), refer to a specific form of lung inflammation characterized by diffuse alveolar damage, noncardiogenic pulmonary edema, as well as pulmonary and systemic neutrophil-associated inflammation [1, 2]. The etiological factors for ALI and ARDS can include various stimuli and diseases such as bacterial and viral pneumonia [3, 4], mechanical ventilation [5, 6], exposure to chemical agents [7, 8], traumatic brain injury [9], sepsis [10, 11], acute pancreatitis [12], and many other pathologies. The recent rise in morbidity and mortality from ALI/ARDS has been associated with the new coronavirus infection (COVID-19) pandemic caused by coronavirus associated with the severe acute respiratory distress syndrome (SARS-CoV-2) [13, 14]. The pathogenesis of ALI/ARDS involves the development of local and systemic cascade inflammatory responses, accompanied by the elevation of the levels of proinflammatory cytokines (TNF-α, IFN-γ, IL-6, IL-1β, GM-CSF, and G-CSF) and chemokines (CXCL10/IP10, MIP-1α, and CCL2) up to critical values, resulting in the development of multiple organ failure [14, 15, 16].

Today, the treatment of ARDS and its accompanying immune disorders is for the most part symptomatic; it aims to alleviate symptoms and often involves mechanical ventilation and the administration of corticosteroids. The use of molecular genetic tools targeting key cytokines can be a promising approach in treating this pathology. Monoclonal antibodies against TNF-α, IL-6, IL-1β, IFN-γ, and other components of cytokine signaling are among such tools [17, 18]. Oligonucleotide aptamers belong to another class of biomolecules that selectively recognize a target and are currently being considered as a potential alternative to antibodies in developing targeted therapeutics. The advantages of aptamers over antibodies include reproducible chemical synthesis and stable key characteristics, as well as the feasibility of making additional chemical modifications in order to control the lifetime of an aptamer in the body while maintaining its affinity for the target molecule [19, 20]. Importantly, aptamers are nucleic acids by their nature, so their functional activity can be further regulated using a complementary nucleotide antidote [21, 22]. Such a set of properties kindles interest in using aptamers to suppress the activity of soluble serum proteins, including inflammation- associated ones [23, 24].

The present study analyzed the features of the development of LPS-induced ALI in mice and the feasibility of suppressing it using the aptamer targeting the proinflammatory cytokine TNF-α. It was demonstrated that TNF-α is a pivotal player in the development of ALI, and that intranasal administration of an aptamer targeting TNF-α, conjugated with 40 kDa polyethylene glycol (PEG-aptTNF-α), to mice with ALI suppresses the development of an inflammation in the respiratory system of experimental animals.

## EXPERIMENTAL

**Synthesis of a TNF-α-targeting DNA aptamer and control oligonucleotide**

**Table 1:** TNF-α-targeting DNA aptamer, non-aptameric scrambled oligodeoxyribonucleotide and their conjugates with PEG

Aptamer	Nucleotide sequence, 5’-3’
PEG-aptTNF-α	PEG-NH_2_-(CH_2_)6-GCG CCA CTA CAG GGG AGC TGC CAT TCG AAT AGG TGG GCC GCT_inv_
aptTNF-α	NH_2_-(CH_2_)6-GCG CCA CTA CAG GGG AGC TGC CAT TCG AAT AGG TGG GCC GCT_inv_
PEG-Scr	PEG-NH_2_-(CH_2_)6-AGA GGC GGT ATG ACC AGG CTA ATC GGC CGA GCC TCC GTG CGT_inv_
Scr	NH_2_-(CH_2_)6-AGA GGC GGT ATG ACC AGG CTA ATC GGC CGA GCC TCC GTG CGT_inv_

PEG – 40 kDa polyethylene glycol residue;

T_inv_ – 3’-terminal residue of thymidine linked to the adjacent nucleotide with a 3’–3’ phosphodiester bond.

*Table 1* lists the nucleotide sequences of the oligodeoxyribonucleotides used in this study. A control random-sequence non-aptameric oligonucleotide was generated on the basis of aptTNF-α DNA aptamer using the service (https://www.genscript.com/tools/create-scrambled-sequence).

The oligonucleotides were synthesized using the solid-phase phosphoramidite method at a scale of 0.4 µmol on an automated ASM-800 DNA/RNA synthesizer (Biosset, Russia) according to the protocol optimized for this setup using β-cyanoethyl- N,N-diisopropyl phosphoramidites of 5’-N-protected 2’-deoxyribonucleosides (Glen Research, USA). CPG (controlled pore glass) particles (pore diameter, 500 Å with 3’-O-dimethoxytrityltimidine attached via the 5’-hydroxyl group (Glen Research) were used as a polymeric carrier. The oligonucleotides containing an aminohexanol residue at the 5’ end were synthesized using a commercially available modifier, 6-(trifluoroacetylamino)-hexyl-(2-cyanoethyl)- (N,N-diisopropyl)-phosphoramidite (Glen Research). After the synthesis, the oligonucleotides were separated from the carrier; the protecting groups were removed by 15-min exposure to a 40% aqueous methylamine solution (300 μL) at 65°C. Fully unblocked oligonucleotides were purified by preparative electrophoresis in a 15% denaturing polyacrylamide gel (PAAG).

**Synthesis of conjugates with 40 kDa polyethylene glycol**

To synthesize PEGylated conjugates, a 1 µmol solution of N-hydroxysuccinimide ester of 40 kDa linear polyethylene glycol (PEG) (Sigma-Aldrich, USA) in dimethylformamide (Sigma-Aldrich) was added to a solution of 5’-amino-modified oligonucleotide (0.1 µmol) in 0.1 M tetraborate buffer (pH 9.5). The reaction mixture was incubated under stirring at 25°C during 16 h. Excess reagents were removed from the resulting conjugates by electrophoresis in denaturing 12% PAAG, followed by elution with water and concentration using Amicon 10K ultracentrifuge modules (Merck, USA). Before being administered to the animals, the purified conjugates were sterilized by passage through a filter (pore diameter, 0.22 µm).

**Laboratory animals**

We used 6- to 8-week-old female Balb/C mice bred in the vivarium of the Institute of Chemical Biology and Fundamental Medicine SB RAS (Novosibirsk, Russia). The animals were housed in a well-lit room (six animals per cage). The mice had free access to food and water. All the manipulations with the animals were performed in accordance with the guidelines for proper use and care of laboratory animals (EU Directive 2010/63/EC). The animal experiments were approved by the Interinstitutional Bioethics Commission of the Institute of Cytology and Genetics SB RAS (Novosibirsk, Russia) (Protocol No. 56 dated August 10, 2019).

**LPS-induced acute lung injury**

Acute lung injury (ALI) was induced in mice by intranasal (i.n.) administration of LPS (055:B5, Sigma- Aldrich) at a dose of 10 µg/mouse under isoflurane anesthesia. In the experiment aiming to study the dynamics of inflammatory changes in the respiratory system, the mice were withdrawn from the experiment 6, 16, and 24 h after induction; bronchoalveolar lavage fluid (BALF), blood serum, and lung tissue samples were collected for further analysis. In the experiment focusing on the anti-inflammatory activity of the TNF-α-targeting aptamers, the apt- TNF-α aptamer at a dose of 1 mg/kg or its conjugate PEG-aptTNF-α at doses of 1 and 5 mg/kg were administered to mice 1 h after i.n. induction of ALI. Mice with ALI, untreated and receiving the respective scrambled oligonucleotides (Scr at a dose of 1 mg/kg and PEG-Scr at doses of 1 and 5 mg/kg), were used as controls. All the agents were administered intranasally in 50 μL of saline under isoflurane anesthesia. The mice were withdrawn from the experiment 24 h after ALI induction, and biomaterial (BALF and lung tissue) samples were collected for further analysis. Each group consisted of six mice.

**Analysis of the bronchoalveolar lavage fluid**

The lungs of the mice in the control and experimental groups were washed with 1 mL of a cold saline solution. The collected BALF samples were centrifuged at 1500 rpm at 4°C during 10 min; the supernatant was collected to conduct an enzyme-linked immunosorbent assay (ELISA). The cell pellet was resuspended in 50 μL of saline; the total leukocyte count (×10^5^ cells/mL) was determined in a Neubauer chamber after 1 : 20 dilution with Türk’s solution.

**Assessment of the levels of proinflammatory cytokines by ELISA**

The levels of the proinflammatory cytokines TNF-α and IL-6 in the BALF samples were quantified using ELISA kits (#BMS607-3 and #KMC0061, Thermo Fisher Scientific, USA) in compliance with the manufacturer’s instructions. Absorbance at 450 nm was measured on a Multiscan RC spectrophotometer (Thermo Labsystems, Finland).

**Assessment of the cytokine profile**

The levels of proinflammatory cytokines and chemokines in the BALF samples were assessed using the LEGENDplexTM Mouse Inflammation Panel (13-plex) (Biolegend, USA) in compliance with the manufacturer’s instructions on a NovoCyte 3000 flow cytometer (ACEA Bioscience, USA). The data were analyzed using the Legendplex online software.

**Histology**

Lung tissue was fixed in 10% buffered formalin, dehydrated in ethanol and xylene solutions of ascending concentration, and embedded into HISTOMIX paraffin. Paraffin sections up to 5 µm thick were cut on a Microm HM 355S microtome and stained with hematoxylin and eosin. All histologic specimens were examined and scanned using an Axiostar Plus microscope equipped with an AxioCam MRc5 digital camera (200× magnification).

The intensity of inflammatory changes in the lungs was assessed semi-quantitatively using the following scale: 0 – no pathological changes; 1 – mild inflammation; 2 – moderate inflammation; and 3 – severe inflammation. A total of five visual fields were analyzed for each sample (30 visual fields in each group).

**Quantification of gene expression levels by real-time RT-PCR**

**Table 2:** Specific primers for real-time RT-PCR

Gene	Primer type	Nucleotide sequence
Usp18	Forward	5’- GCCCTCATGGTCTGGTTG-3’
	Probe	5’-((5,6)-FAM)-ACGTGTTGCCTTAACTCCTTGCTTCA-BHQ1-3’
	Reverse	5’- CACTTCTCTTCCTCTCTTCTGC-3’
Traf1	Forward	5’- AGATCACCAATGTCACCAAGC-3’
	Probe	5’-((5,6)-FAM)-ACTGTCAGCCTCTTCTCTCCAGCTT-BHQ1-3’
	Reverse	5’- CATCCCCGTTCAGGTACAAG-3’
Tnfaip3	Forward	5’- AGCCAGCACTTTGTACCC-3’
	Probe	5’-((5,6)-FAM)-AGTCTTCAAACCTACCCCGGTCTCT-BHQ1-3’
	Reverse	5’- GCTTTTCCTTCATCTCATTCTCAG-5’
Hprt	Forward	5’-CCCCAAAATGGTTAAGGTTGC-3’
	Probe	5’- ((5,6)-ROX)-CTTGCTGGTGAAAAGGACCT-3’-BHQ2
	Reverse	5’-AACAAAGTCTGGCCTGTATCC-3’

The mRNA levels of the *Usp18*, *Traf1*, *Tnfaip3*, and* Hprt *genes in lung tissue were determined by quantitative real-time reverse transcription polymerase chain reaction (RT-PCR). Total RNA was isolated from the lungs of the experimental animals using the TRIzol reagent according to the manufacturer’s instructions after pre-homogenization (FastPrep- 24TM 5G homogenizer equipped with a QuickPrep 24 adapter, MP Biomedicals, USA). cDNA was synthesized using RT buffer and M-MuLV-RH revertase (Biolabmix, Russia) according to the manufacturer’s instructions. Real-time RT-PCR was carried out using the HS-qPCR master mix (×2) (Biolabmix) according to the manufacturer’s instructions. Amplification was performed under the following temperature conditions: (1) 94°C, 5 min; (2) 94°C, 10 s; and (3) 60°C, 30 s (50 cycles) on a C1000 Touch amplifier with a CFX96 module (Bio-Rad, USA). Data were processed using the BioRad CFX Manager software. *Table 2* lists the sequences of oligonucleotides used as primers. The relative gene expression level was normalized to the level of *Hprt *expression using the ΔΔCt method.

**Statistical analysis**

Statistical analysis was conducted using the two-tailed unpaired Student’s t-test in the Microsoft Excel software. The p ≤0.05 values were considered statistically significant. Data are presented as the mean value ± standard deviation.

## RESULTS

**The cytokine profile and the dynamics of inflammatory changes in the respiratory system of mice with LPS-induced acute lung injury**

**Fig. 1:**
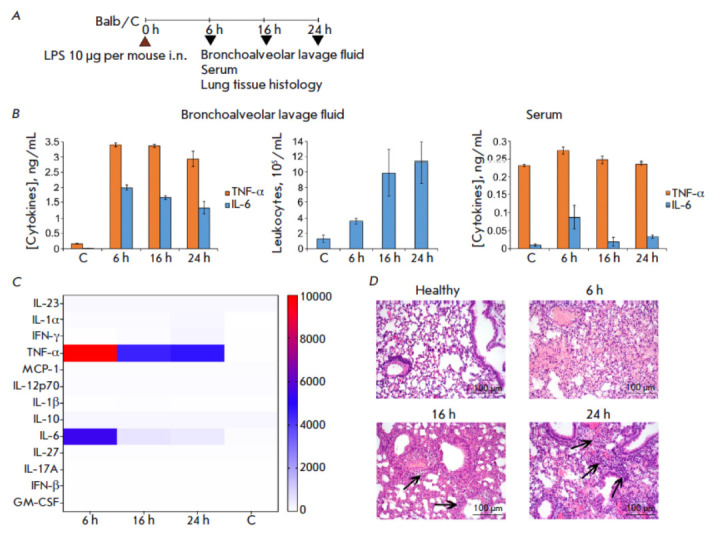
The cytokine profile and inflammatory changes in the respiratory system of mice with LPS-induced acute lung injury over time. (*A*) Experimental design. Acute lung injury (ALI) was induced in Balb/C mice by intranasal (i.n.) administration of LPS (10 μg/mouse). Material was collected 6, 16, and 24 h after induction. (*B*) The levels of proinflammatory cytokines (TNF-α and IL-6) and the total leukocyte count in BALF, as well as serum levels of TNF-α and IL-6 in ALI mice 6, 16, and 24 h after induction. (*C*) The cytokine profile of BALF of ALI mice over time, assessed using multiplex ELISA. Data are presented as pg/mL. (*D*) Histological analysis of the lung tissue of ALI mice 6, 16, and 24 h after induction. Hematoxylin and eosin staining, original magnification 200×. Black arrows indicate inflammatory infiltration. C – control (healthy animals)

For assessing the cytokine level and the intensity of the respiratory system inflammation, acute lung injury (ALI) was induced in mice by intranasal (i.n.) administration of LPS (10 μg/mouse), followed by the sampling of material 6, 16, and 24 h after induction (*Fig. 1A*). As early as 6 h after LPS administration, the TNF-α level in the bronchoalveolar lavage fluid (BALF) had increased 21-fold compared with that in the healthy animals and was 3.4 ng/mL, remaining approximately unchanged until 24 h after ALI induction. The IL-6 levels had also increased to 2 ng/mL 6 h after LPS administration and then gradually decreased to 1.3 ng/mL by the time point of 24 h post-induction, being manifold higher than the IL-6 levels in the healthy animals (*Fig. 1B*, left panel). Assessment of the total leukocyte count in BALF showed that the maximum value of this parameter was observed 24 h post-induction: LPS administration increased leukocyte count 8.6-fold compared with that in the healthy animals (*Fig. 1B*, middle panel). Extremely low levels of TNF-α and IL-6 were detected in the serum samples of mice with ALI at all time points, being comparable to those in the healthy animals (*Fig. 1B*, right panel).

Identification of the cytokine profiles of BALF in mice with ALI over time by multiplex ELISA showed that i.n. administration of LPS significantly increased the levels of only two proinflammatory cytokines: TNF-α and IL-6. The TNF-α level remained high throughout the entire observation period, whereas the IL-6 level had declined already 16 h after ALI induction (*Fig. 1C*). The levels of other cytokines (IL-23, IL-1α, IFN-γ, MCP-1, IL-12p70, IL-1β, IL-10, IL-27, IL-17A, IFN-β, and GM-CSF) remained unchanged.

Histological analysis of the lung tissue samples from mice with ALI showed that LPS administration induced pathological changes in the respiratory system manifesting themselves as inflammatory granulocyte infiltration, destructive (desquamation of alveolar lining) and dyscirculatory (congestion, edema and hemorrhage) disorders (*Fig. 1D*). The severity of these changes varied at different time points throughout the observation period: changes associated with circulatory disturbances were prevailing 6 h after ALI induction; after 16 h, there appeared initial signs of cell migration to the inflammation site; whereas by 24 h after ALI induction, inflammatory infiltration in the lung tissue had fully taken hold, mainly being located around vessels and bronchi (*Fig. 1D*).

Hence, assessing the dynamics of inflammatory changes and the cytokine profile in mice with ALI showed that the TNF-α level remained steadily high throughout the entire observation period, thus indicating that this cytokine plays a crucial role in the signaling pathways of the inflammatory response. The LPS-induced morphological signs of inflammation in the respiratory system of mice were most intense 24 h after induction, making this time interval optimal for assessing the anti-inflammatory activity of the tested constructs.

**The choice and synthesis of the anti-TNF-α DNA aptamer**

Several nucleotide sequences of RNA and DNA aptamers capable of specifically binding TNF-α have been reported [25]. Most of them have affinity for the target protein in the nanomolar range and can inhibit the functional activity of TNF-α *in vitro*. The apt- TNF-α DNA aptamer, which had been earlier shown to be able to suppress the development of inflammation in *in vivo *models of acute lung and liver injury when administered intravenously or intratracheally, was chosen for the study [26]. The total length of this aptameric oligonucleotide is 41 nucleotides, which makes its chemical synthesis rather fast and economically sound (the sequence of aptTNF-α is listed in *Table 1*). A scrambled oligonucleotide having the same length and nucleotide composition was used as a similar non-aptameric DNA to control the specificity of aptamer activity. The *in vivo *anti-inflammatory activity of bivalent aptamers in which two aptamer modules are covalently linked by a 20 kDa polyethylene glycol residue has been demonstrated earlier [26]. In our study, we chose a different strategy to chemically modify the aptamer in order to increase its lifetime in the animal body and used modifications that are currently almost the “gold standard” for aptamers intended for use in *in vivo *experiments or clinical trials [27, 28]. An additional thymidine residue linked by the 3’–’ phosphodiester bond was inserted into the 3’ end to prevent exonuclease hydrolysis; the commercially available polymer-bound 3’-O-dimethoxytritylthymidine was used in solid- phase oligonucleotide synthesis for this purpose, and 40 kDa polyethylene glycol (PEG) was inserted at the 5’ end to improve the pharmacokinetic characteristics of the aptamer being topically delivered into the respiratory system of mice.

Therefore, the proposed aptamer variant ensures better synthesis control and isolation of the PEGylated conjugate, since at the detected TNF-α levels in BALF (≤ 4 ng/mL), this protein is most likely to exist in a monomeric form rather than a trimeric one [29]. A similar set of modifications was also used for the control non-aptameric oligonucleotide.

**Anti-inflammatory activity of the TNF-α-targeting aptamer in the model of LPS-induced acute lung injury**

**Fig. 2:**
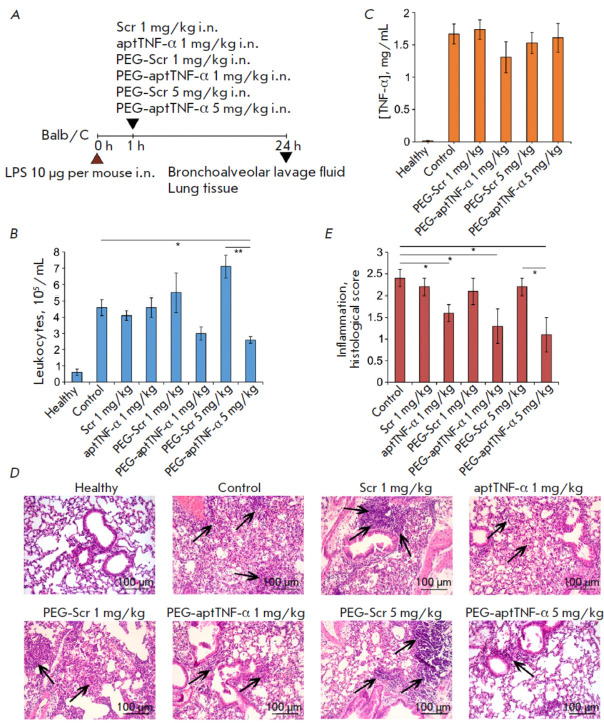
The effect of TNF-α-targeting aptamers on the development of LPS-induced acute lung injury in mice. (*A*) Experimental design. Acute lung injury (ALI) was induced in Balb/C mice by intranasal (i.n.) administration of LPS (10 μg/mouse). Mice were administered i.n. TNF-α-targeting aptamers: aptTNF-α at a dose of 1 mg/kg and PEG-aptTNF-α at doses of 1 and 5 mg/kg 1 h after induction. Mice with ALI without treatment and treated with the scrambled oligonucleotide (Scr and PEG-Scr) were used as controls. Mice were sacrificed 24 h after induction, and material was collected for subsequent analysis. (*B*, *C*) The total number of leukocytes (*B*) and TNF-α level (*C*) in the bronchoalveolar lavage fluid (BALF) of mice with LPS-induced ALI without treatment and after administration of TNF-α-targeting aptamers. (*D*, *E*) Histological examination (*D*) and semi-quantitative assessment of the intensity of the inflammatory changes (*E*) in the lungs of mice in the control and experimental groups. Hematoxylin and eosin staining, original magnification 200×. Black arrows indicate inflammatory infiltration. The following scale was used to assess inflammation in the lungs: 0 – no pathological changes; 1 – mild inflammation; 2 – moderate inflammation; and 3 – severe inflammatory changes. Data are presented as the mean ± standard deviation; *p  < 0.05, **p  < 0.01

The anti-inflammatory activity of TNF-α-targeting aptamers (aptTNF-α and PEG-aptTNF-α) was studied in the model of LPS-induced ALI. Aptamers were administered intranasally (i.n.) to mice, since high levels of this cytokine were specifically detected in BALF. Respective scrambled oligonucleotides (Scr or PEG-Scr) were administered to mice in order to control the specificity of aptamer activity. The studied constructs were administered 1 h after ALI induction, followed by sampling of the material 24 h later (*Fig. 2A*), since it was the time point when morphological changes in the respiratory system of the mice were the most intense and complete.

Administration of LPS increased the total leukocyte count in the BALF of mice with ALI 7.7-fold compared to that in the healthy animals (*Fig. 2B*). Administration of aptTNF-α or the respective Scr had no effect on this parameter, whereas 5 mg/kg of the PEGylated aptamer PEG-aptTNF-α statistically significantly reduced the total leukocyte count in BALF 1.8-fold vs. control and 2.7-fold vs. PEG-Scr administered at the same dose (*Fig. 2B*). PEG-aptTNF-α administered at a dose of 1 mg/kg also caused a 1.5- and 1.8-fold drop in this parameter compared to that observed for the control and Scr, respectively. These differences (for the dose of 1 mg/kg) were statistically insignificant; however, they showed that the activity of PEG-aptTNF-α was dose-dependent. The TNF-α level in the BALF of mice with ALI was elevated 85-fold compared to that in the healthy animals, and administration of PEG-aptTNF-α had no significant effect on this parameter (*Fig. 2C*). The TNF-α level after administration of the non-PEGylated aptamer was not investigated, since it did not reduce the total leukocyte count in BALF as an indicator of the antiinflammatory activity of the drug.

According to histological findings, administration of the constructs under study reduces the intensity of morphological manifestations of the LPS-induced ALI described previously in a dose-dependent manner. Administration of aptTNF-α and PEG-aptTNF-α at a dose of 1 mg/kg decreased the intensity of inflammatory changes in lung tissue 1.5- and 1.8-fold compared to that in the control group and 1.4- and 1.6-fold compared to the respective Scr (*Fig. 2D,E*). However, these differences were statistically significant only when comparing the aptamers and the controls. Administration of 5 mg/kg PEG-aptTNF-α led to a statistically significant decline in the inflammation intensity in the lung tissue compared both with the control group (2.2-fold) and the group receiving PEG-Scr (2-fold).

Hence, the PEG-aptTNF-α aptamer targeting proinflammatory TNF-α cytokine suppresses the development of LPS-induced inflammatory changes in the respiratory system of mice but does not normalize the parameters to the level of healthy animals. The antiinflammatory activity of aptTNF-α/PEG-aptTNF-α is dose-dependent, which is most likely related to the fact that TNF-α binding increases with the preparation dose, while the anti-inflammatory effect was reliably demonstrated only for the conjugate with PEG, due to the improved pharmacokinetic characteristics of the agent.

**Analysis of the expression levels of TNF-α-regulated genes in the lung tissue of mice with LPS-induced acute lung injury without treatment and after administration of the TNF-α-targeting aptamer**

The next stage of our study involved a search for and assessment of the expression levels of potential TNF-α-regulated genes upon the development of LPS-induced ALI and its correction with the TNF-α-targeting aptamer to seek confirmation that the constructs used affect the ability of the secreted target protein to bind to its receptor and activate sig naling. The genes (namely, *Usp18*, *Traf1, *and *Tnfaip3*) were chosen based on the published data according to which their expression levels are upregulated by TNF-α in a broad range of biological and pathological processes, such as LPS-induced sepsis [30], myocardial ischemia reperfusion injury [31], cerebral ischemia [32], activation of the NF-κB and type I interferon- mediated signaling pathways [33, 34, 35, 36], as well as hematopoiesis and regeneration of the myeloid lineage [37].

**Fig. 3:**
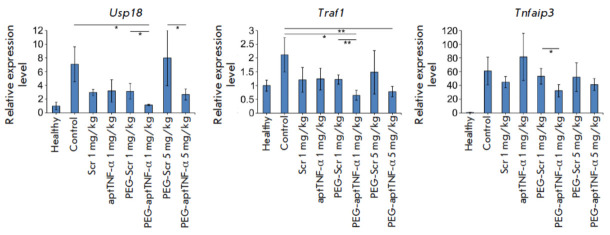
The expression levels of potential target genes of TNF-α (*Usp18*, *Traf1*, and *Thfaip3*) in the lung tissue of mice with LPS-induced acute lung injury without treatment and after administration of aptamers. Gene expression levels were normalized to the expression level of *Hprt*, which was used as an internal standard. Three samples from each group were analyzed in triplicate. Data are presented as the mean ± standard deviation; *p  < 0.05, **p  < 0.01

The expression levels of the *Usp18*, *Traf1*, and* Tnfaip3 *genes in the lung tissue of mice were assessed by RT-PCR (*Fig. 3*). The lungs of healthy animals were characterized by low expression levels of the genes under study (assumed equal to unity (1)), whereas administration of LPS significantly increased their expression levels (control): *Usp18*, sevenfold;* Traf1*, twofold; and *Tnfaip3*, 61-fold compared to those in healthy animals.

The following patterns were revealed when assessing the effect of aptamers on the expression of the studied genes in the lung tissue of mice with ALI. Administration of PEG-aptTNF-α at a dose of 1 mg/kg led to a statistically significant decline in the *Usp18 *gene expression level to a level typical of healthy animals: 5.9-fold vs. control and 2.6-fold vs. PEG-Scr. PEG-aptTNF-α administered at a dose of 5 mg/kg also resulted in a 2.6- and 3-fold decrease in the *Usp18 *expression level compared to the control and PEG-Scr groups, respectively. However, statistically significant differences were revealed only between the aptamer and scrambled oligonucleotide (*Fig. 3*). Administration of PEG-aptTNF-α at doses of 1 and 5 mg/kg caused a 3.5- and 2.6-fold decrease in* Traf1 *gene expression compared with the control and 1.8- and 1.7-fold decrease compared with PEG-Scr, respectively. However, no statistically significant differences in the effects of PEG-aptTNF-α and PEG-Scr at a dose of 5 mg/kg were observed (*Fig. 3*). For the* Tnfaip3 *gene, the only statistically significant difference was the 1.6-fold decrease in the expression level upon exposure to 1 mg/kg PEG-aptTNF-α vs. the PEG-Scr group (*Fig. 3*). Administration of PEG-Scr at doses of 1 and 5 mg/kg had no statistically significant effect on the expression of all the studied genes. Administration of 1 mg/kg aptTNF-α resulted in a statistically insignificant 1.7- to 2.4-fold decline in the expression levels of the *Usp18 *and *Traf1 *genes and a 1.3-fold increase in expression vs. the control group in the case of the *Tnfaip3* gene (*Fig. 3*).

Hence, the most prominent decline in the expression levels of potential target genes was observed when administering 1 mg/kg PEG-aptTNF-α, whereas significant anti-inflammatory effects were detected when this aptamer was administered at a dose of 5 mg/kg, which may be related to the choice of time points to quantify the expression levels of these genes, when the maximum effect of a higher aptamer dose on the expression of TNF-α-associated genes has already passed.

## DISCUSSION

TNF-α, a pleiotropic cytokine produced by activated macrophages, T cells, and natural killer cells, is among the most important immune response regulators; therefore, affecting the level of this cytokine can be an efficient strategy for correcting immune disorders associated with cancer, as well as inflammatory, metabolic, and infectious diseases [38, 39]. Taking into account the variety of diseases and biological processes involving TNF-α, several dozen genes and signaling pathways regulate TNF-α and are regulated by it [40, 41].

Today, monoclonal antibodies are the key anti- TNF-α drugs approved for clinical use; however, they cause adverse events such as increased susceptibility to infections, as well as the development of demyelinating diseases and malignancies [41]. Anti-TNF-α agents of a different nature, such as aptamers, may possibly reduce the rate and severity of complications.

In this study, the anti-inflammatory activity of the TNF-α-targeting aptamer, as well as the effect of chemical modifications on its effectiveness, was studied in the mouse model of LPS-induced ALI. The choice of the aptamer administration route was justified and verified by the cytokine profile data in BALF and serum samples upon development of ALI in mice over time. The TNF-α level in BALF was shown to be significantly higher than that in serum, thus indicating that topical intranasal administration of the aptamer is more promising compared to its systemic administration. As for the aptamer doses used in the experiment, we chose concentrations that were likely to ensure the target effect taking into account that PEGylated aptamer concentrations in the range of 1–10 mg/kg are optimal for *in vivo *experiments and clinical studies [42].

PEG-aptTNF-α was found to exhibit a stronger anti-inflammatory effect than its unmodified analog, which may be due to its longer lifetime in the animal body. PEG-aptTNF-α was dose-dependent: at a dose of 5 mg/kg, its effectiveness was higher compared to a dose of 1 mg/kg. However, despite the anti-inflammatory activity observed in BALF and lung tissue, no significant decline in the TNF-α level was detected in BALF. This discrepancy can result from the fact that the aptamer and anti-TNF-α antibodies used in ELISA may bind to different spatially distant epitopes of TNF-α, interaction with the aptamer having no effect on antibody binding. However, while no data obtained in structural or molecular modeling studies are available that would indicate to which particular TNF-α epitope anti-TNF-α aptamer binds, this hypothesis requires further verification.

Since direct measurements of the TNF-α level failed to yield the anticipated results, we decided to assess the effect of the TNF-α-targeting aptamer by analyzing the mRNA expression level of the genes involved in the regulation and transduction of TNF-α signaling. Genes directly involved in the TNF-α regulatory pathway, whose expression level increases during the development of ALI, were chosen as potential TNF-α-regulated genes.

**Fig. 4:**
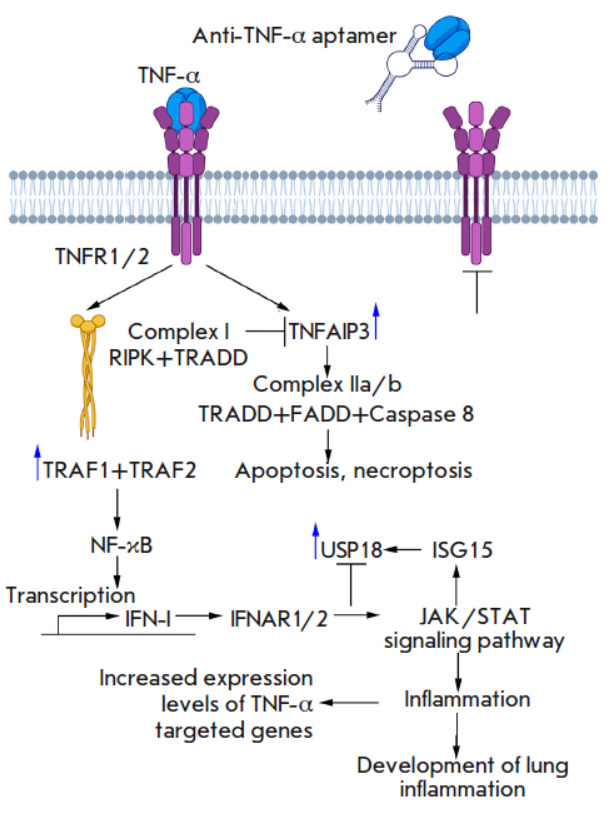
The general scheme of TNF-α signaling

In the early phase of signaling, the soluble form of TNF-α binds to the tumor necrosis factor receptor 1 (TNFR1), causing receptor trimerization and involvement of the TNFR1-associated death domain protein (TRADD) and receptor-interacting serine/threonine protein kinase 1 (RIPK1) (*Fig. 4*). Next, TRADD interacts with the TRAF1/TRAF2 heterodimer to form complex I, which activates the NF-κB signaling pathway and induces the synthesis of proinflammatory cytokines, including IFN-I [33]. Through IFNAR1/2 receptors and the JAK/STAT signaling pathway, IFN-I has a further impact on the next important component of the development of the inflammatory response: the ISG15/USP18 axis, which regulates the activity of the immune system [36] and reduces the inflammatory response intensity by inhibiting the JAK/STAT signaling pathway, indicating that there possibly exists a negative feedback loop between USP18, IFN-I, and, therefore, TNF-α [43, 44].

TNF-α-induced protein 3 (TNFAIP3), also known as A20, is another key molecule in the mechanism of reverse regulation. The basal level of TNFAIP3 expression is low in most cells but increases rapidly as the inflammatory response develops [45]. TNFAIP3 is recruited to the TNFR1 signaling complex, where it deubiquitinates RIPK1, thus resulting in a loss of stability by complex I and inhibits further activation of NF-κB. TRADD dissociated from complex I forms complexes with the Fas-associated death domain protein (FADD) and caspase 8 (complex IIa) or with RIPK1, FADD, and caspase 8 (complex IIb), which further leads to apoptosis or necroptosis [46].

Taking into account the involvement of the aforementioned genes in TNF-α signaling and regulation, as expected, administration of the TNF-α-targeting aptamer reduced the expression level of the target genes chosen for validation (namely, *Traf1 *as a component of the heterodimeric complex, which is directly involved in TNF-α signal transduction; *Tnfaip3*, which inhibits the activation of the proinflammatory NF-κB signaling pathway; and *Usp18*, which regulates the intensity of the inflammatory response after NF-κB activation via the negative feedback mechanism), suggesting that it is reasonable to use TNF-α- regulated genes to assess the biological activity of the anti-TNF-α aptamer. Similar to the case with morphological changes in the respiratory system of mice, PEG-aptTNF-α exhibited a stronger effect on gene expression levels than unmodified aptamers did.

Hence, this study once again demonstrated the importance of TNF-α as a therapeutic target in ALI, as well as the benefits of using chemically modified aptamers to suppress its function. A secretory protein is a very attractive target for an aptamer, since the agent does not need to be delivered into the cell for binding to it; instead, a therapeutic aptamer can be systemically or locally administered to organs and tissues where target protein levels are elevated. Furthermore, it is very encouraging that the aptamer can be administered after the onset of the pathology and that its activity can be inhibited with an antidote, which makes therapeutic aptamers almost “ideal drugs.”

## CONCLUSIONS

It has been found in this study that TNF-α is among the pivotal players in cytokine signaling during the development of LPS-induced ALI and that intranasal administration of anti-TNF-α aptamers efficiently mitigates the LPS-induced inflammatory changes in the respiratory system of mice, affects the TNF-α-regulated genes, and can be viewed as a tool for treating ALI of different etiologies and other pulmonary diseases accompanied by immune disorders.

## Abbreviations

ALI: acute lung injury
ARDS: acute respiratory distress syndrome
TNF-α: tumor necrosis factor α
aptTNF-α: TNF-α-targeting aptamer
PEG: polyethylene glycol
LPS: lipopolysaccharide
BALF: bronchoalveolar lavage fluid
